# 

**Published:** 1906-07

**Authors:** 


					﻿SUBSCRIPTION $1.00
ATLANTA "y'iww*
JOURNAL-RE(ffla)
“'OJF MEDICINE.
i
Successor to Atlanta rtf dical and Surgical Journal, Established 1855,
and Southern Hedical Record, Established 1870.
Vol. VIII.	Atlanta, Ga., July, 1906.	No. 4. ...
				

